# Influences of Sample Preparation on Nanoindentation Behavior of a Zr-Based Bulk Metallic Glass

**DOI:** 10.3390/ma5061033

**Published:** 2012-06-01

**Authors:** Hu Huang, Hongwei Zhao, Zhiyu Zhang, Zhaojun Yang, Zhichao Ma

**Affiliations:** 1 College of Mechanical Science & Engineering, Jilin University, Renmin Street 5988, Changchun 130025, China; E-Mails: huanghuzy@163.com (H.H.); yzj@jlu.edu.cn (Z.Y.); 455090724@qq.com (Z.M.); 2 Key Laboratory of Optical System Advanced Manufacturing Technology, Chinese Academy of Science, Changchun 130033, China; E-Mail: zhangzhiyu126@126.com

**Keywords:** mechanical polishing, plunge cutting, nanoindentation, serrated flow, creep

## Abstract

Influences of two different sample preparation methods, mechanical polishing and plunge cutting, on nanoindentation behavior of a Zr-based bulk metallic glass were studied. Mechanical polishing suppresses the serrated flow but promotes the creep. In contrast, plunge cutting promotes the serrated flow but suppresses the creep. However, hardness and elastic modulus obtained from these two methods are nearly the same.

## 1. Introduction

Due to unique mechanical properties such as high strength, modulus, hardness and elastic limit [1,2,3,4,5], bulk metallic glasses (BMGs) are considered as emerging structural materials since their discovery in 1960 [6,7]. However, BMGs are relatively brittle compared to their crystalline counterparts at room temperature, and the poor ductility and formation of shear bands induced by highly localized inhomogeneous deformation limit BMGs applications [8,9]. Formation of shear bands reduces the strength of BMGs, which leads to brittle fractures along the shear planes [10]. So, mechanical properties and the deformation mechanism of BMGs have been given more and more attention. Although compression and tensile testing of BMGs can now be carried out, instrumented indentation, especially nanoindentation, remains as an important tool to study mechanical properties of BMGs because of its high spatial and temporal resolution [11]. Via nanoindentation, researchers have done many interesting works. Occurrence of the serrated flow and formation of shear bands underneath indents have been reported during indentation tests [12,13,14,15]. The serrated flow means that the load-displacement curve has regions of sudden displacement increase, without any corresponding increase of the load. In 2003, Schuh [12] reported that the serrated flow corresponds to activation of individual shear bands, and that it is strongly dependent on the indentation loading rate—slower indentation loading rates promote more conspicuous serrations, and rapid indentations suppress the serrated flow. There is a critical applied strain rate for each kind of BMGs, above which the serrated flow is completely suppressed. Similar conclusions were obtained by other researchers [16,17]. In 2004, Schuh [18] studied effects of both temperature and the loading rate on the serrated flow and attained a quantitative framework for their theory.

Previous researchers mainly focused on influences of the loading rate [16], the strain rate [17] and temperature [15] on the mechanical behavior of BMGs. As is well known, the pop-in phenomenon which is similar with the serrated flow has been observed in numerous materials, but occurrence of pop-in can be dramatically reduced by mechanically altering the surface. Many researchers have confirmed this conclusion. Oliver and Pharr [19] reported that while pop-in occurs regularly on an electropolished tungsten surface, it is entirely eliminated when the surface is prepared by mechanical polishing. Lucca *et al.* [20] showed that pop-in is suppressed in ZnO single crystals by mechanical polishing with 1/4 μm and 1 μm diamond abrasives. Wang *et al.* [21] undertook a systematic study of effects of mechanical polishing, chemo-mechanical polishing and electropolishing on pop-in behavior of single-crystal Mo, and they found that the pop-in phenomenon is extremely sensitive to surface preparation details. In this paper, we will study influences of mechanical polishing and plunge cutting on nanoindentation behavior of a Zr-based bulk metallic glass, and evaluate the effects of different sample preparation methods on the serrated flow and creep of the bulk metallic glass.

## 2. Materials and Experiments

**Figure 1:**
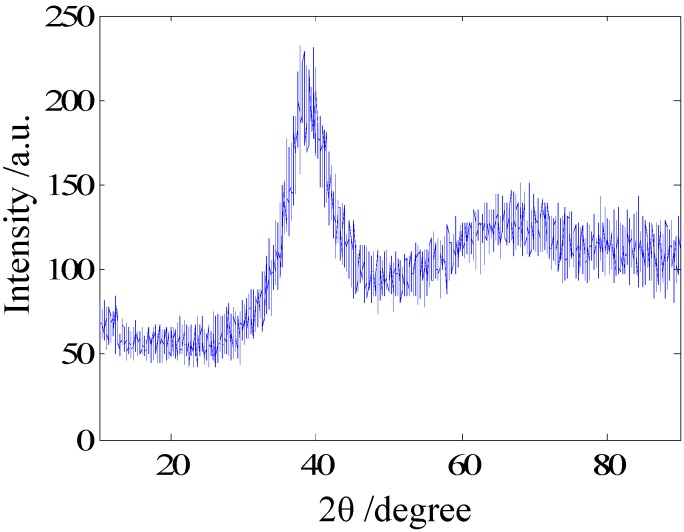
XRD pattern of the as-cast Zr_46.5_Cu_45_Al_7_Ti_1.5_ alloy.

Alloy ingots with a nominal composition of Zr_46.5_Cu_45_Al_7_Ti_1.5_ in atomic percent were produced by arc-melting the mixtures of Zr, Cu, Al, and Ti elements in a Ti-gettered high-purity argon atmosphere, and more information about the BMG can be found in the literature [22]. The amorphous nature of the BMG was verified by an X-ray diffractometer (XRD, Cu, *K*_α_ radiation). The result of the XRD is presented in Figure 1. There is only a broad diffraction peak, which corresponds to an amorphous structure. No peaks associated with any crystalline phase can be observed.

Firstly, mechanical polishing with 1.3 μm Al_2_O_3_ abrasive was carried out to prepare the sample surface. After polishing, the nanoindentation test by a Vickers indenter was conducted with the maximum load of 10 mN. In order to observe the serrated flow and study the influences of sample preparation on the serrated flow, a low load rate 0.04 mN/s was selected according to previous literatures [16,17]. The dwell time of 30 s was set to study the influence on creep. Then, plunge cutting was used to process the sample surface. Plunge cutting was carried out on an ultraprecision machine tool (AHN-05, JTEKT Corporation, Osaka, Japan), having a linear positioning resolution of 1 nm. A round-nosed single-crystal diamond tool with tool edge radius of 50 nm was used in the process. Depth of cutting was set to 1 µm at a constant cutting speed of 500 mm/min (0.0083 m/s). At such a low cutting speed, effects of cutting heat generation will be insignificant. Cutting oil (Bluebe #LB10) was used in the form of mist jet. Using the above-mentioned experimental conditions, a surface roughness (Ra < 5 nm) could be generated, which is suitable for nanoindentation tests. Using the same indenter and experimental parameters, the nanoindentation test was carried out on the plunge cutting surface.

## 3. Results and Discussion

**Figure 2:**
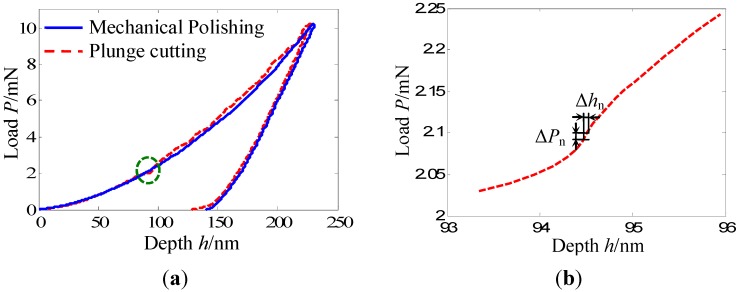
(**a**) Typical load-depth (*P*-*h*) curves of the Zr-based bulk metallic glass under different sample preparation methods; (**b**) Partial enlarged view of (**a**) highlighting details of the serrated flow.

The load-depth (*P*-*h*) curves of these two experiments are shown in Figure 2a. On the whole, these two curves are coincident well with each other, especially the unloading portions.

According to the method of Oliver and Pharr [19], main mechanical parameters such as hardness and elastic modulus, which are related with the unloading portions of the *P*-*h* curves, are nearly the same. That is to say, these two sample preparation methods, mechanical polishing and plunge cutting, almost have no affect on the hardness and elastic modulus of the BMG. However, an obvious difference is observed from the loading portions. A more conspicuous serration flow appears in the loading portion of the *P*-*h* curve obtained from the plunge cutting surface. Figure 2b shows a partially enlarged view of Figure 2a, highlighting details of the serrated flow. The loading portion of the *P*-*h* curve obtained from the mechanical polishing is relatively smooth. More detailed analysis, as follows, highlights this difference.

Define Δ*h*_n_ and Δ*P*_n_ as the depth difference and load difference of the n-th sampling point (*h*_n_, *P*_n_) and the previous sampling point (*h*_n−1_, *P*_n−1_) respectively. So, Δ*h*_n_ and Δ*P*_n_ can be expressed as:

Δ*h*_1_ = *h*_1_ (1)

Δ*h*_n_ = *h*_n_ − *h*_n−1_ (n = 2, 3…) (2)

Δ*P*_1_ = *P*_1_ (3)

Δ*P*_n_ = *P*_n_ − *P*_n−1_, (n = 2, 3…) (4)

Because indentation experiments were carried out with the same loading rate and sampling rate, the load difference Δ*P*_n_ is a constant but the depth difference Δ*h*_n_ will change with the increase of the penetration depth. Especially, when the serrated flow appears in the *P*-*h* curve, for example in the loading portion as shown in Figure 2b, the value of the depth difference Δ*h*_n_ will increase quickly and then decrease quickly with the increase of the penetration depth. Thus, a peak, which can be used to clearly identify the serrated flow, will appear. Height and width of peaks demonstrate the size of the serrated flow. Higher and wider peaks express larger and more conspicuous serrated flow.

**Figure 3:**
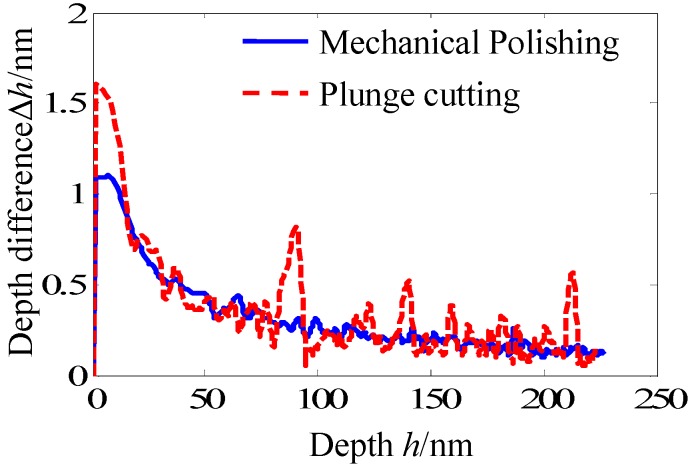
Depth difference Δ*h*_n_ as a function of the penetration depth under different sample preparation methods.

Figure 3 shows the depth difference Δ*h*_n_ as a function of the penetration depth. Because the indentation experiments were carried out with a constant loading rate, the depth difference Δ*h*_n_ in the small depth region were larger than that in the large depth region. With the increase of the penetration depth, obvious peaks are observed from the curve of the plunge cutting surface. However, the curve of the mechanical polishing surface is relatively smooth. The number, height and width of peaks of the plunge cutting surface are larger than that of the mechanical polishing surface. So, the conclusion can be obtained that plunge cutting promotes more conspicuous serrations and mechanical polishing suppresses the serrated flow. In addition, position of the serrated flow can be easily determined from Figure 3.

**Figure 4:**
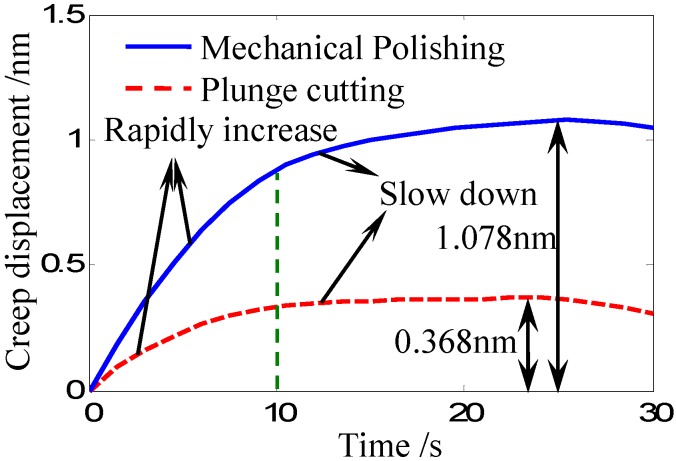
Creep displacement curves of the Zr-based bulk metallic glass during the dwell time under different sample preparation methods.

The recorded creep displacement curves of the Zr-based bulk metallic glass during the dwell time under different sample preparation methods are shown in Figure 4. During the dwell time, the creep displacement is observed to increase rapidly at the beginning of the dwell time, and then slow down and trend to be stable. It can also be seen that, creep displacement of the mechanical polishing surface is obviously larger than that of the plunge cutting surface. The maximum creep displacement of the mechanical polishing surface is about 1.078 nm while the maximum value of the plunge cutting surface is about 0.368 nm. Figure 4 shows that different sample preparation methods result in different creep rates. Mechanical polishing promotes the creep while plunge cutting suppresses the creep. Although the creep rates are different under different sample preparation methods, the transition point between the rapidly increase section and the slow down section is nearly the same at the timing point of 10 second.

## 4. Conclusions

Experimental results in this paper show that the two different sample preparation methods, mechanical polishing and plunge cutting, significantly affect the serrated flow and creep of the Zr-based bulk metallic glass. Mechanical polishing suppresses the serrated flow, but promotes the creep. In contrast, plunge cutting promotes the serrated flow, but suppresses the creep. However, hardness and elastic modulus obtained are nearly the same. The reason for these differing behaviors may be that different preparation methods induce different deformed layers because of dissimilar machining mechanism. The exact nature of these differences will be carefully studied by cross-sectional transmission electron microscope in the future. However, whatever the mechanism, the following two recommendations can be offered. First, a suitable and uniform sample preparation method should be established for mechanical testing of BMGs to maintain the uniformity and comparability of the experimental data because it affects the mechanical behavior of BMGs. Second, suitable manufacturing methods and processes should be selected for the application of BMGs, because they affect the mechanical properties, especially the serrated flow and formation of shear bands, which increase the fatigue and damage of BMGs.

## References

[1] Greer A.L. (1995). Metallic glasses. Science.

[2] Inoue A. (2000). Stabilization of metallic supercooled liquid and bulk amorphous alloys. Acta Mater..

[3] Inoue A., Shen B.L., Koshiba H., Kato H., Yavari A.R. (2004). Ultra-high strength above 5000 MPa and soft magnetic properties of Co-Fe-Ta-B bulk glassy alloys. Acta Mater..

[4] Wang W.H., Dong C., Shek C.H. (2004). Bulk metallic glasses. Mater. Sci. Eng. Rep..

[5] Scully J.R., Gebert A., Payer J.H. (2007). Corrosion and related mechanical properties of bulk metallic glasses. J. Mater. Res..

[6] Duwez P., Wilens R.H., Klement W. (1960). Continuous series of metastable solid solutions in silver-copper alloys. J. Appl. Phys..

[7] Johnson W.L. (2002). Bulk amorphous metal—An emerging engineering material. JOM J. Miner. Met. Mater..

[8] Johnson W.L. (1999). Bulk glass-forming metallic alloys: Science and technology. Mater. Res. Soc. Bull..

[9] Eckert J., Das J., Pauly S., Duhamel C. (2007). Mechanical properties of bulk metallic glasses and composites. J. Mater. Res..

[10] Xing D., Zhang T., Li W., Wei B. (2007). The characterization of plastic flow in three different bulk metallic glass systems. J. Alloy. Compd..

[11] Schuh C.A., Nieh T.G. (2004). A survey of instrumented indentation studies on metallic glasses. J. Mater. Res..

[12] Schuh C.A., Nieh T.G. (2003). A nanoindentation study of serrated flow in bulk metallic glasses. Acta Mater..

[13] Wang J.G., Choi B.W., Nieh T.G., Liu C.T. (2000). Crystallization and nanoindentation behavior of a bulk Zr-Al-Ti-Cu-Ni amorphous alloy. J. Mater. Res..

[14] Yoo B.G., Oh J.H., Kim Y.J., Jang J.I. (2010). Effect of hydrogen on subsurface deformation during indentation of a bulk metallic glass. Intermetallics.

[15] Li N., Liu L., Chan K.C., Chen Q., Pan J. (2009). Deformation behavior and indentation size effect of Au_49_Ag_5.5_Pd_2.3_Cu_26.9_Si_16.3_ bulk metallic glass at elevated temperatures. Intermetallics.

[16] Chen K.W., Jian S.R., Wei P.J., Jang J.S.C., Lin J.F. (2010). The study of loading rate effect of a Cu-based bulk metallic glass during nanoindentation. J. Alloy. Compd..

[17] Yoo B.G., Lee K.W., Jang J.I. (2009). Instrumented indentation of a Pd-based bulk metallic glass: Constant loading-rate test *vs* constant strain-rate test. J. Alloy. Compd..

[18] Schuh C.A., Lund A.C., Nieh T.G. (2004). New regime of homogeneous flow in the deformation map of metallic glasses: Elevated temperature nanoindentation experiments and mechanistic modeling. Acta Mater..

[19] Oliver W.C., Pharr G.M. (1992). An improved technique for determining hardness and elastic modulus using load and displacement sensing indentation experiments. J. Mater. Res..

[20] Lucca D.A., Klopfstein M.J., Ghisleni R., Cantwell G. (2002). Investigation of polished single crystal ZnO by nanoindentation. CIRP Ann. Manuf. Technol..

[21] Wang Z.G., Bei H., George E.P., Pharr G.M. (2011). Influences of surface preparation on nanoindentation pop-in in single-crystal Mo. Scr. Mater..

[22] Chen L.Y., Xue Z., Xu Z.J., Chen J.Q., He R.X., Nie X.P., Cao Q.P., Wang X.D., Ding S.Q., Jiang J.Z. (2011). Cu-Zr-Al-Ti bulkmetallic glass with enhanced glass-forming ability, mechanical properties, corrosion resistance and biocompatibility. Adv. Eng. Mater..

